# Perinatal Mortality in Sub-Saharan Africa: A Meta-Analysis of Demographic and Health Surveys

**DOI:** 10.5334/aogh.2348

**Published:** 2019-07-12

**Authors:** Blessing Jaka Akombi, Andre Masumbuko Renzaho

**Affiliations:** 1 School of Social Sciences and Psychology, Western Sydney University, AU; 2 School of Public Health and Community Medicine, University of New South Wales, AU

## Abstract

**Background:**

Sub-Saharan Africa (SSA) has one of the highest levels of perinatal mortality globally. However, there are sub-regional and country-specific disparities in its distribution.

**Objective:**

The aim of this study was to undertake a meta-analysis of demographic and health surveys to quantify perinatal mortality rate within sub-Saharan Africa and to depict sub-regional and country-specific differences.

**Methods:**

This study used cross-sectional data from the most recent demographic and health surveys (2010–2016) conducted in 21 sub-Saharan African countries. The countries were grouped into four sub-regions (Eastern Africa, Western Africa, Southern Africa and Central Africa), and a meta-analysis was conducted to estimate perinatal mortality rate within each of the sub-regions. Significant heterogeneity was detected among the various surveys (I^2^ > 50%), hence a random effect model was used. Sensitivity analysis was also performed to examine the effects of outliers. Perinatal mortality was defined as pregnancy losses occurring after seven completed months of gestation (stillbirths) and deaths to live births within the first seven days of life (early neonatal deaths).

**Findings:**

The pooled estimate for perinatal mortality rate per 1000 births across 21 countries in the four sub-regions of SSA was 34.7 (95% CI: 32.6, 36.8). Eastern Africa reported 34.5 (95% CI: 32.2, 36.8), with the highest rate observed in Tanzania [39.5 (95% CI: 35.8, 43.4)]. Western Africa reported 35.7 (95% CI: 32.2, 39.3), with the highest rate observed in Nigeria [40.9 (95% CI: 38.3, 43.2)]. Southern Africa reported 30.3 (95% CI: 26.5, 34.0), with the highest rate observed in Lesotho [49.6 (95% CI: 42.3, 57.8)]. Central Africa reported 30.7 (95% CI: 28.0, 33.3), with the highest rate observed in Equatorial Guinea [37.3 (95% CI: 30.5, 45.1)].

**Conclusions:**

To reduce mortality in the perinatal period, interventions should focus on improving access to high quality antenatal and postnatal care, as well as strengthening health care systems within countries in sub-Saharan Africa.

## Background

Perinatal deaths are pregnancy losses occurring after 28 weeks of gestation (stillbirths) and deaths to live births within the first seven days of life (early neonatal deaths) [1]. These untimely deaths are a major public health problem in many developing countries and have enormous economic, social and health implications for families and society [2]. The distinction between a stillbirth and an early neonatal death may be a fine one, which often depends on observing the faint signs of life post-delivery [3]. Unlike neonatal mortality that only accounts for deaths to live births, perinatal mortality also accounts for stillbirths, making it a comprehensive indicator for estimating the true level of mortality around the time of delivery [3,4]. The causes of stillbirths and early neonatal deaths are interlinked and reflect the quality of obstetric and paediatric care available during pregnancy and delivery, as well as post-delivery. Perinatal mortality occurs due to a complex interaction of individual level factors relating to maternal lifestyle and maternal obstetric complications, which could be exacerbated by underlying community-level factors, such as lack of access to good quality maternal and newborn health services, to clean water supply, to proper antenatal and postnatal nutrition for the mother and the newborn and poor environmental sanitation, as well as societal factors relating to political instability and armed conflicts [1].

Globally, the average stillbirth rate per 1000 total births declined from 24.7 in 2000 to 18.4 in 2015 [5], and neonatal mortality rate also fell from 37 deaths per 1000 live births in 1990 to 19 in 2016 [6]. Consequently, the number of perinatal deaths decreased from 5.7 million in 2000 to 4.1 million in 2015 [7], with 95% of these untimely deaths occurring in South Asia and sub-Saharan Africa (SSA) [6,7]. The decline in perinatal mortality, though slower than that reported in child mortality [6], is an indication of the increased attention being given to improving newborn health and preventing stillbirths. Further significant progress is envisaged with the launch of the Every Newborn Action Plan (ENAP), the United Nation (UN) Secretary-General’s Every Woman Every Child (EWEC) monitoring framework, the “Every Child Alive” campaign, spearheaded by UNICEF, and the Quality of Care network, by the UN Inter-Agency Group for Mortality Estimation (IGME) 2017, all of which are focused on reducing perinatal mortality in low and middle income (LMIC) countries and achieving the Sustainable Development Goals (SDGs).

As emphasised in the recent WHO 2018 Progress Report “Reaching every newborn national 2020 milestones”, there is a need for target-setting, particularly to reduce perinatal mortality rates within South Asia and SSA. To achieve this goal, a clear knowledge of the distribution of perinatal mortality across SSA countries is essential to inform prioritization of regional initiatives, which focus on encouraging national-level advocacy and effective interventions that strengthen the health system of high burden countries. Previous studies have reported the burden and determinants of stillbirth [8,9,10] and neonatal mortality [11,12,13] across SSA; however, no study has critically analysed the pooled rate of perinatal mortality within the World Health Organization (WHO) geographical sub-regions of SSA, Western Africa, Eastern Africa, Central Africa and Southern Africa, to inform sub-regional comparisons. Hence, the main aim of this study was to conduct a meta-analysis of perinatal mortality in SSA using the most recent nationally representative demographic and health surveys (DHS) from 23 SSA countries.

## Method

### Data sources

The data analysed in this study were extracted from the most recent DHS (2010–2016) of 21 SSA countries. The datasets are publicly available from the DHS website [14]. The DHS are nationally representative and population-based surveys with a high response rate (>90%). The surveys have large sample sizes (usually between 5000 and 30,000 households) and are conducted about every 5 years to allow comparisons over time. DHS collate data that are comparable across countries using standardized methods involving uniform questionnaires, manuals and field procedures. The surveys use a stratified, multi-stage (cluster), random sampling design and are conducted using 5 questionnaires: the household questionnaire, the woman’s questionnaire, the man’s questionnaire, the biomarker questionnaire, the fieldworker questionnaire, and the verbal autopsy questionnaire (for neonatal deaths). In all households, women and men aged 15 to 49 years are eligible to participate. Details on data collection and sampling methodology employed by DHS are described elsewhere [14].

### Mortality indicators

Stillbirth was defined as foetal deaths in pregnancies lasting 7 or more months. Early neonatal deaths were defined as deaths at age 0 to 6 days among live-born children. Perinatal mortality rate is the sum of the number of stillbirths and early neonatal deaths divided by the number of pregnancies of 7 or more months’ duration, expressed per 1000.

### Inclusion criteria

**Figure 1:**
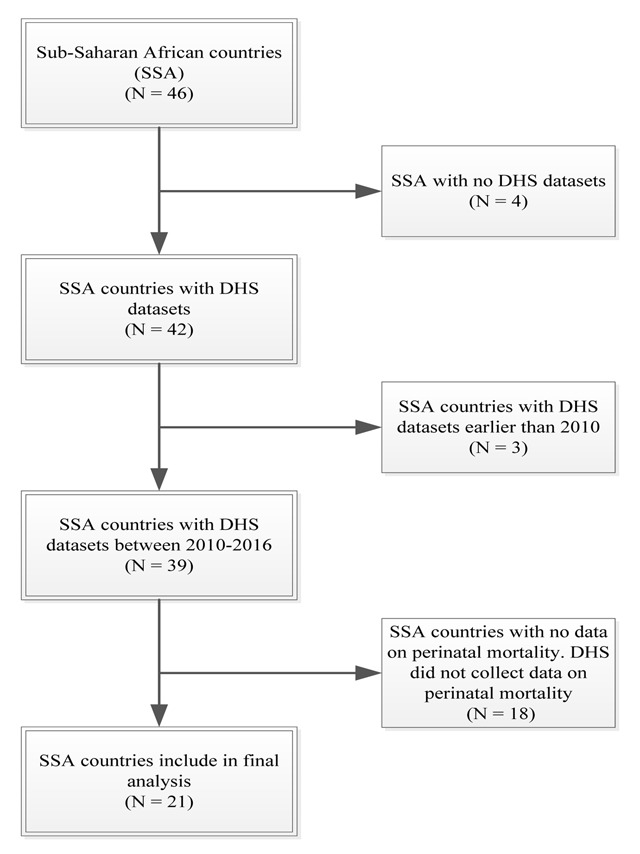
Flow chart for country selection.

Countries with recent DHS (2010–2016) and comprehensive data on perinatal mortality (number of stillbirths, number of early neonatal deaths and number of pregnancies of 7+ months) were included in this study. Countries with DHS from 2010 to 2016 were eligible for inclusion to capture a more recent burden of perinatal mortality and to inform policy prioritization. Additionally, it serves as a needs assessment for countries without recent DHS datasets. The flowchart for country selection based on the inclusion criteria is presented in Figure 1. The country classification was based on UN geoscheme classification for SSA.

Table 1 presents the 21 countries included in this study, the year of DHS, their respective sub-regions, the number of stillbirths, the number of early neonatal deaths and the number of pregnancies of 7+ months.

**Table 1:** Countries of the different sub-regions in SSA and their mortality indicators.

Country	Year of DHS	Number of stillbirths	Number of early neonatal deaths	Number of pregnancies of 7+ months’

***Eastern Africa***				
Burundi	2016	313	215	13835
Ethiopia	2016	130	236	11071
Kenya	2014	126	146	9484
Malawi	2015	236	378	17485
Mozambique	2011	127	320	11831
Rwanda	2014	125	114	8129
Uganda	2016	251	330	15437
Tanzania	2015	187	214	10163
Zambia	2013	180	247	13563
Zimbabwe	2015	77	140	6437
***Western Africa***				
Gambia	2013	89	151	7995
Ghana	2014	81	140	5776
Liberia	2013	70	129	6572
Niger	2012	225	229	13571
Nigeria	2013	396	925	32224
Senegal	2016	133	102	6206
Sierra Leone	2013	100	377	12298
***Southern Africa***				
Lesotho	2014	74	84	3184
Namibia	2013	39	77	4843
***Central Africa***				
Angola	2015	104	292	13380
Equatorial Guinea	2011	22	79	2709

### Data analysis

This study was based on secondary data analysis. The number of stillbirths, early neonatal deaths and pregnancies of ≥7 months were extracted from the respective country DHS. These data were used to estimate perinatal mortality rate for each country. The syntax “metaprop” in Stata version 14.0 (StataCorp, College Station, TX, USA) was used to generate forest plots of perinatal mortality rate in each included country, its corresponding weight, as well as the pooled rate across each sub-region and its corresponding 95% confidence intervals (Cl). A test of heterogeneity of the data obtained for the different countries showed a high level of inconsistency (I^2^ > 50%) for countries in Eastern Africa and Western Africa, thus a random effect model was used in all the meta-analysis. Sensitivity analyses were conducted using similar methods adopted by Patsopoulos and colleagues [15], which involves comparing the pooled rate before and after eliminating countries with outliers one at a time. The percentage of observed total variation across countries (I^2^) was not computed for Southern and Central Africa due to low sample size.

## Results

**Figure 2:**
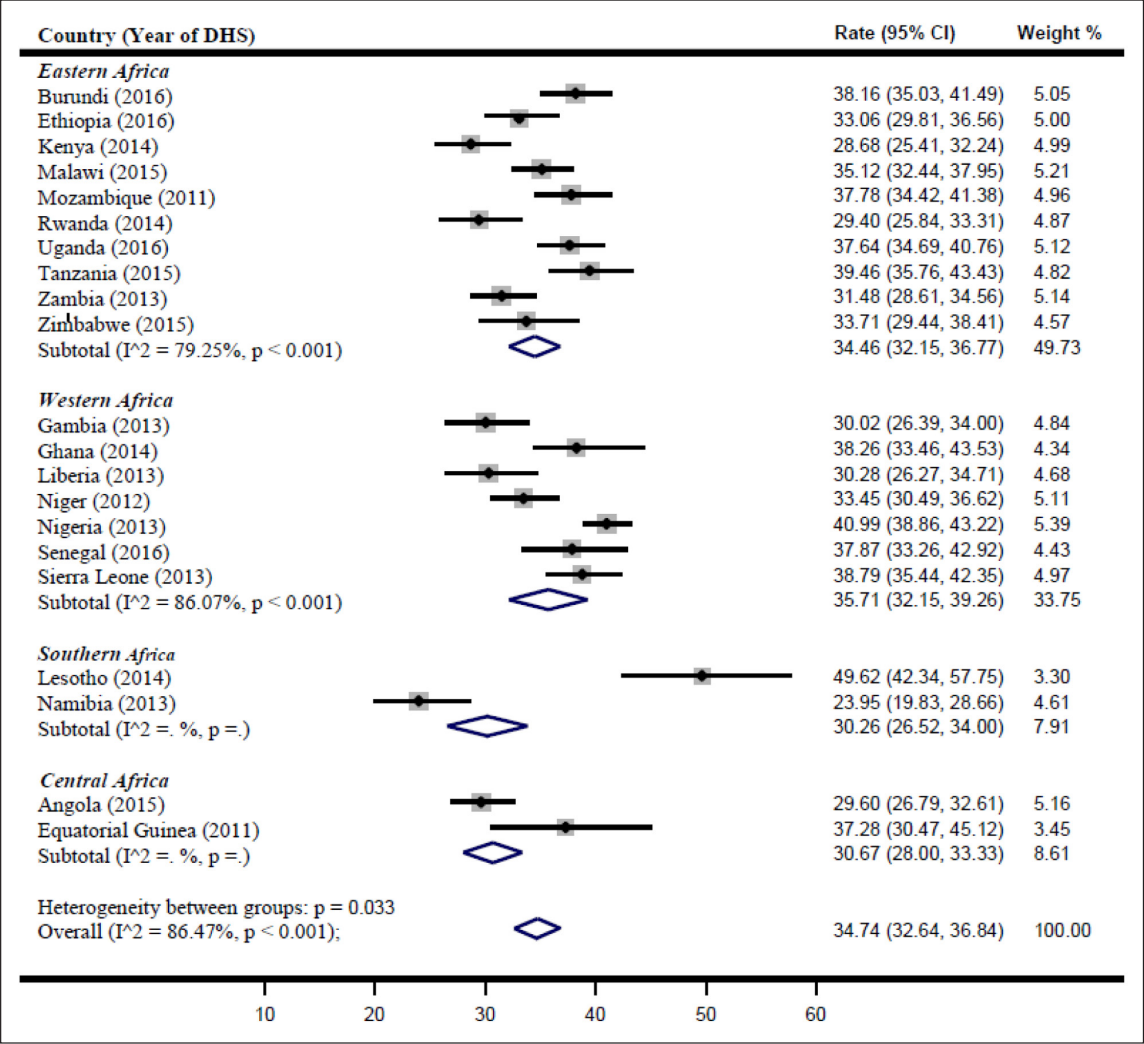
Perinatal mortality rate per 1000 births in the 4 sub-regions of SSA.

Figure 2 presents a forest plot of perinatal mortality rate per 1000 births in the 4 sub-regions of SSA. The pooled rate across 21 countries in SSA was 34.7 (95% CI: 32.6, 36.8).

### Eastern Africa

The pooled estimate of perinatal mortality rate for Eastern Africa was 34.5 (95% CI: 32.2, 36.8). Tanzania (39.5 [95% CI: 35.8, 43.4]), Burundi (38.2 [95% CI: 35.0, 41.5]) and Mozambique (37.8 [95% CI: 34.4, 41.4]) reported the highest perinatal mortality rate across the sub-region. However, the rate reported was not significant because their confidence interval overlapped with the pooled sub-regional estimate. While Kenya (28.7 [95% CI: 25.4, 32.1]) reported the lowest perinatal mortality rate in the sub-region.

### Western Africa

The pooled estimate of perinatal mortality rate for Western Africa was 35.7 (95% CI: 32.2, 39.3). Nigeria (40.9 [95% CI: 38.3, 43.2]) reported the highest perinatal mortality rate within the region. Sierra Leone (38.8 [95% CI: 35.4, 42.4]), Ghana (38.3 [95% CI: 33.5, 43.5]) and Senegal (37.9 [95% CI: 33.3, 42.9]) also reported high perinatal mortality rate within the sub-region, though the rate were not significant.

### Southern Africa

The pooled estimate of perinatal mortality rate for Southern Africa was 30.3 (95% CI: 26.5, 34.0). Lesotho reported a perinatal mortality rate of 49.6 (95% CI: 42.3, 57.8), while Namibia reported 24 (95% CI: 20, 29). Lesotho also reported the highest perinatal mortality rate across the 21 SSA countries analysed.

### Central Africa

The pooled estimate of perinatal mortality rate for Central Africa was 30.7 (95% CI: 28.0, 33.3). Of the two countries analysed in the sub-region, Equatorial Guinea (37.3 [95% CI: 30.5, 45.1]) reported a higher rate of perinatal mortality.

## Discussion

The estimate for perinatal mortality within SSA was 34.7 per 1000 births. The pooled sub-regional estimates for Eastern, Western, Southern and Central Africa were 34.5, 35.7, 30.3 and 30.7 per 1000 births, respectively. Exploring perinatal mortality on a sub-regional level provides a closer view into the inter-country differences in the burden of disease in order to promote policy prioritization in high burden countries. Countries within each sub-region have experienced socio-political, economic and cultural events that could influence child survival differently. Countries such as Lesotho in Southern Africa, Nigeria in West Africa, Tanzania in East Africa and Equatorial Guinea in Central Africa reported the highest perinatal mortality rate within each sub-region.

Developing countries have reported higher perinatal mortality rate globally [1]. The situation is particularly critical within sub-regions in SSA where most countries have a poor health service delivery system due to conflict, lack of political commitment and a weak health care system [16,17]. Countries in SSA have experienced conflicts in recent years. Studies have shown that conflict serves as a barrier to facility-based deliveries and often results in the destruction of health infrastructure and a lack of skilled health personnel, leading to an increase in the risk of perinatal mortality due to unassisted births and a lack of timely provision of life saving emergency perinatal services [18,19]. To address the health inequality caused by conflict within SSA countries, there is a need for proper health infrastructure, equipment, medicines and skilled health personnel.

In this study, Lesotho reported the highest rate of perinatal mortality in SSA. Though Lesotho has achieved great strides in improving its health indicators [20], the country has also experienced high perinatal mortality rate, which might be due to the high HIV prevalence in the country [21]. Lesotho has one of the highest rates for HIV globally and the second highest in SSA [22]. The interaction of HIV with other maternal infections during pregnancy results in poor birth outcomes [23] as pregnancy accelerates the progression of infections due to its immunosuppressive effect [24]. Studies have shown that maternal HIV status leads to an increased risk of stillbirth and death in the neonatal period [23,24,25]. Studies have also shown that HIV-positive mothers are more likely to have low birth weight neonates than HIV-negative mothers [23,26,27], with low birth weight being an established risk factor for neonatal deaths [28,29,30]. However, it is worth noting Namibia and Swaziland also have high HIV prevalence but have maintained a lower perinatal mortality rate. Hence, there is a need for further research into the reason for the higher perinatal mortality observed in Lesotho to inform targeted interventions.

In this study, Nigeria reported the highest perinatal mortality rate in West Africa and the second highest in SSA. With 68% of the population living on less than $1.25 per day and 86.9 million of its 180 million inhabitants living in extreme poverty [31,32], the high level of perinatal mortality in Nigeria could be a result of poverty in the country. Poverty remains an underlying factor associated with poor birth outcomes, and studies have shown a significant association between poverty and perinatal mortality [33,34]. Poverty is multidimensional, with economic, political, social, governance, health and environmental components that influence child survival at the individual, household and societal level [35,36]. Poverty results in the inability to afford health care costs, which leads to poor utilization of health care services, thus increasing the risk of perinatal mortality. Therefore, addressing poverty with a target to reduce child mortality, especially in the perinatal period, is critical to improving child survival among vulnerable sub-populations in Nigeria and SSA.

SSA has experienced climate change, which is associated with an increased incidence of vector-borne infectious diseases, such as malaria [37]. The risk of malaria has been reported as high across countries in all sub-regions of SSA and has been exacerbated by human factors [38]. Malaria infection during pregnancy is a major public health problem in SSA and is known to be associated with higher rates of stillbirth, premature delivery, low-birth-weight neonates and neonatal death [39]. The link between malaria and perinatal mortality has been widely researched [39,40,41]. A systematic review of 117 studies reported a higher perinatal mortality rate in malaria endemic countries than in non-endemic countries [42]. However, malaria-induced perinatal deaths have declined in high burden countries within SSA due to current preventative measures, such as providing pregnant women with insecticide-treated bed nets (ITN) and intermittent preventive treatment (IPT) with antimalarial medications [43]. WHO recommends that women receive at least 3 doses of IPT with sulfadoxine-pyrimethamine (SP) during pregnancy, with each dose being given at least 1 month apart starting from the second trimester and safely administered up until the time of delivery. This will result in further decline in perinatal mortality in all areas with moderate to high malaria transmission in SSA [44].

Other unfavourable climatic conditions, such as droughts, are frequent and severe in many countries of Eastern and Southern Africa [45]. During drought periods, water becomes scarce and pregnant women are most likely to resort to unsafe water sources, making them more vulnerable to waterborne diseases, such as cholera and diarrhoea, which are known causes of perinatal mortality [46,47,48,49]. Additionally, during periods of droughts, food production is greatly affected, which could lead to malnutrition—an indirect cause of perinatal mortality [50]. Previous studies conducted on the impact of maternal nutrition on birth outcomes reported suboptimal maternal nutrition results in intra-uterine growth restriction (IUGR) and low birth weight, which are also known risk factors for perinatal mortality [50,51,52].

In general, addressing perinatal mortality within the sub-regions of SSA would involve coordinated national and international efforts by governments and non-governmental organisations, as well as strengthening individual countries healthcare systems. The removal of financial barriers to the utilization of healthcare services during pregnancy and facility-based deliveries would improve access to quality antenatal, delivery and postnatal care. Additionally, the inclusion of a strong newborn component in emergency preparedness and response plans for countries experiencing political instability, conflicts, climate change and other potentially destabilizing conditions is highly needed.

This study has a number of strengths. First, the DHS are nationally representative and population based with a large sample size. Second, this study showed the distribution of perinatal mortality across countries in SSA to inform regional policy prioritization among high burden countries. However, the study was limited as some SSA countries were not included because they had no recent DHS data (2010–2016) or had no comprehensive data on the perinatal mortality indicators. Second, only two countries in Central Africa met the inclusion criteria; hence, the pooled estimate for Central Africa might not be representative of the sub-region. Third, the test of heterogeneity for Southern and Central Africa yielded no estimate due to low sample size within these sub-regions; hence, we are unable to establish true heterogeneity between the countries in Southern and Central Africa. Furthermore, most of the explanations provided for the higher rate of perinatal mortality in certain countries are shared across SSA countries; hence, more research is needed to ascertain the country-specific reasons for the higher rates observed.

## Conclusion

This study reveals the countries within SSA with the highest perinatal mortality rates. To address perinatal mortality in SSA, strategies should focus on community-based mobilization of women to seek adequate antenatal care services and facility-based deliveries, as well as improved nutrition, up-to-date immunizations and healthy water, sanitation and hygiene practices. These interventions will reduce perinatal mortality in SSA, thereby setting the region on the path to achieving the SDG target by 2030.

## References

[1] World Health Organization. Neonatal and perinatal mortality: Country, regional and global estimates Geneva, Switzerland: World Health Organization; 2006.

[2] World Health Organization. 2018 Progress Report: Reaching every newborn national 2020 milestones World Health Organization; 2018 3.

[3] National Population Commission [Nigeria] and ICF International. Nigeria Demographic and Health Survey 2013 Abuja, Nigeria and Rockville, Maryland, USA: NPC and ICF International; 2014.

[4] Chinkhumba J, De Allegri M, Muula AS and Robberstad B. Maternal and perinatal mortality by place of delivery in sub-Saharan Africa: A meta-analysis of population-based cohort studies. BMC Public Health. 2014 12; 14(1): 1014 DOI: 10.1186/1471-2458-14-1014 25263746 PMC4194414

[5] Bernis L, Kinney MV, Stones W, et al. Stillbirths: Ending preventable deaths by 2030. The Lancet. 2016; 387(10019): 703–716. DOI: 10.1016/S0140-6736(15)00954-X 26794079

[6] UNICEF, WHO, World Bank Group and United Nations. Levels and Trends in Child Mortality Report 2017 UNICEF; 2017 10.

[7] Wang H, Bhutta ZA, Coates MM, et al. Global, regional, national, and selected subnational levels of stillbirths, neonatal, infant, and under-5 mortality, 1980–2015: A systematic analysis for the Global Burden of Disease Study 2015. The Lancet. 388(10053): 1725–1774. 10.1016/S0140-6736(16)31575-6 PMC5224696 27733285

[8] Stringer EM, Vwalika B, Killam WP, et al. Determinants of stillbirth in Zambia. Obstetrics & Gynecology. 2011 5 1; 117(5): 1151–9. DOI: 10.1097/AOG.0b013e3182167627 21508755

[9] Berhie KA and Gebresilassie HG. Logistic regression analysis on the determinants of stillbirth in Ethiopia. Maternal Health, Neonatology and Perinatology. 2016 12; 2(1): 10 DOI: 10.1186/s40748-016-0038-5 PMC5025573 27660718

[10] Dahiru T and Aliyu AA. Stillbirth in Nigeria: Rates and risk factors based on 2013 Nigeria DHS. Open Access Library Journal. 2016 8 30; 3(08): 1 DOI: 10.4236/oalib.1102747

[11] Grady SC, Frake AN, Zhang Q, et al. Neonatal mortality in East Africa and West Africa: A geographic analysis of district-level demographic and health survey data. Geospatial Health. 2017 5 26; 12(1). DOI: 10.4081/gh.2017.501 28555482

[12] Kananura RM, Tetui M, Mutebi A, et al. The neonatal mortality and its determinants in rural communities of Eastern Uganda. Reproductive Health. 2016 12; 13(1): 13 DOI: 10.1186/s12978-016-0119-y 26883425 PMC4756421

[13] Kayode GA, Ansah E, Agyepong IA, Amoakoh-Coleman M, Grobbee DE and Klipstein-Grobusch K. Individual and community determinants of neonatal mortality in Ghana: A multilevel analysis. BMC Pregnancy and Childbirth. 2014 12; 14(1): 165 DOI: 10.1186/1471-2393-14-165 24884759 PMC4036104

[14] The DHS Program. [Cited; [http://dhsprogram.com/data/data-collection.cfm].

[15] Patsopoulos NA, Evangelou E and Ioannidis JP. Sensitivity of between-study heterogeneity in meta-analysis: Proposed metrics and empirical evaluation. International Journal of Epidemiology. 2008 4 18; 37(5): 1148–57. DOI: 10.1093/ije/dyn065 18424475 PMC6281381

[16] Kirigia J and Barry S. Health challenges in Africa and the way forward. International Archives of Medicine. 2008; 1: 27 DOI: 10.1186/1755-7682-1-27 19094201 PMC2615747

[17] Prata N, Passano P, Rowen T, Bell S, Walsh J and Potts M. Where there are (few) skilled birth attendants. Journal of Health, Population, and Nutrition. 2011 4; 29(2): 81 DOI: 10.3329/jhpn.v29i2.7812 PMC3126980 21608417

[18] Paxton A, Maine D, Freedman L, Fry D and Lobis S. The evidence for emergency obstetric care. International Journal of Gynecology & Obstetrics. 2005 2 1; 88(2): 181–93. DOI: 10.1016/j.ijgo.2004.11.026 15694106

[19] UNICEF. The State of the World’s Children 2012: Children in an urban world New York: UNICEF; 2012 2.

[20] Mwase T, Kariisa E, Doherty J, Hoohlo-Khotle N, Kiwanuka-Mukiibi P and Williamson T. Lesotho health systems assessment 2010 Bethesda, MD: Health Systems 2010; 20: 20.

[21] Government of Lesotho. Health Sector Strategic Plan 2012/13-2016/17 Maseru: Government of Lesotho; 2013 4.

[22] UNAIDS. AIDSinfo. http://aidsinfo.unaids.org/. Accessed Month Day, Year.

[23] Temmerman M, Plummer FA, Mirza NB, et al. Infection with HIV as a risk factor for adverse obstetrical outcome. AIDS (London, England). 1990 11; 4(11): 1087–93. DOI: 10.1097/00002030-199011000-00006 2282181

[24] Ryder RW and Temmerman M. The effect of HIV-1 infection during pregnancy and the perinatal period on maternal and child health in Africa. Aids. 1991; 5: S75–85. 1669928

[25] Francisca M, Dorothee EN, Anne-Cecile ZK, David C and Ekoe T. HIV exposure and related newborn morbidity and mortality in the University Teaching Hospital of Yaoundé, Cameroon. Pan African Medical Journal. 2011 4 16; 8(43). DOI: 10.4314/pamj.v8i1.71160 PMC3201607 22121451

[26] Van de Perreí P. Perinatal transmission of HIV-1: Lack of impact of maternal HIV infection on characteristics of livebirths and on neonatal mortality in Kigali, Rwanda. Aids. 1991; 5(3): 295–300. DOI: 10.1097/00002030-199103000-00008 2059369

[27] Ezeaka VC, Iroha EO, Akinsulie AO, Temiye EO and Adetifa IM. Anthropometric indices of infants born to HIV-1-infected mothers: A prospective cohort study in Lagos, Nigeria. International Journal of STD & AIDS. 2009 8; 20(8): 545–8. DOI: 10.1258/ijsa.2008.008446 19625585

[28] Lau C, Ambalavanan N, Chakraborty H, Wingate MS and Carlo WA. Extremely low birth weight and infant mortality rates in the United States. Pediatrics. 2013 5 1; 131(5): 855–60. DOI: 10.1542/peds.2012-2471 23545381

[29] Alexander GR, Kogan M, Bader D, Carlo W, Allen M and Mor J. US birth weight/gestational age-specific neonatal mortality: 1995–1997 rates for whites, Hispanics, and blacks. Pediatrics. 2003 1 1; 111(1): e61–6. DOI: 10.1542/peds.111.1.e61 12509596 PMC1382183

[30] Lawn JE, Cousens S, Zupan J, Lancet Neonatal Survival Steering Team. 4 million neonatal deaths: When? Where? Why? The Lancet. 2005 3 5; 365(9462): 891–900. DOI: 10.1016/S0140-6736(05)71048-5 15752534

[31] World Bank. World Development Indicators 2013 Washington, D.C.: World Bank http://data.worldbank.org/data-catalog/world-development-indicators. Accessed August 2018.

[32] Hunger Notes. World Hunger and Poverty Facts and Statistics; 2016 http://www.worldhunger.org/articles/Learn/world%20hunger%20facts.

[33] Mmusi-Phetoe RM. Social factors determining maternal and neonatal mortality in South Africa: A qualitative study. Curationis. 2016; 39(1): 1–8. DOI: 10.4102/curationis.v39i1.1571 PMC6091558 27381720

[34] Ghimire PR, Agho KE, Akombi BJ, et al. Perinatal mortality in South Asia: Systematic review of observational studies. International Journal of Environmental Research and Public Health. 2018 7; 15(7). DOI: 10.3390/ijerph15071428 PMC6069066 29986453

[35] Mohanty SK. Multidimensional poverty and child survival in India. PLoS One. 2011 10 27; 6(10): e26857 DOI: 10.1371/journal.pone.0026857 22046384 PMC3203176

[36] Lang VF and Lingnau H. Defining and measuring poverty and inequality post-2015. Journal of International Development. 2015 4; 27(3): 399–414. DOI: 10.1002/jid.3084

[37] UNICEF. State of the World’s Children 2016: A fair chance for every child UNICEF; 2016 https://www.unicef.org/publications/files/UNICEF_SOWC_2016.pdf.

[38] Mouchet JE, Manguin S, Sircoulon JA, et al. Evolution of malaria in Africa for the past 40 years: Impact of climatic and human factors. Journal of the American Mosquito Control Association. 1998 6; 14(2): 121–30. 9673911

[39] Schantz-Dunn J and Nour NM. Malaria and pregnancy: A global health perspective. Reviews in Obstetrics and Gynecology. 2009; 2(3): 186. 19826576 PMC2760896

[40] Menéndez C, Bardaji A, Sigauque B, et al. Malaria prevention with IPTp during pregnancy reduces neonatal mortality. PLoS One. 2010 2 26; 5(2): e9438 DOI: 10.1371/journal.pone.0009438 20195472 PMC2829080

[41] Taha TE and Gray RH. Malaria and perinatal mortality in central Sudan. American Journal of Epidemiology. 1993 10 15; 138(8): 563–8. DOI: 10.1093/oxfordjournals.aje.a116896 8237979

[42] Van Geertruyden JP, Thomas F, Erhart A and D’alessandro UM. The contribution of malaria in pregnancy to perinatal mortality. The American Journal of Tropical Medicine and Hygiene. 2004 8 1; 71(2_suppl): 35–40. DOI: 10.4269/ajtmh.2004.71.35 15331817

[43] World Health Organization. World Malaria Report 2015 WHO; 2015 12.

[44] World Health Organization. Malaria: Intermittent preventive treatment in pregnancy (IPTp) WHO Updated 2018 6 21 http://www.who.int/malaria/areas/preventive_therapies/pregnancy/en/.

[45] Benson C and Clay E. Drought and Sub-Saharan African Economies. Africa Region Findings & Good Practice Infobriefs; No. 118 World Bank, Washington, DC: World Bank; 1998 https://openknowledge.worldbank.org/handle/10986/9884 License: CC BY 3.0 IGO. DOI: 10.1596/0-8213-4180-4

[46] Tran NT, Taylor R, Antierens A and Staderini N. Cholera in pregnancy: A systematic review and meta-analysis of fetal, neonatal, and maternal mortality. PloS One. 2015 7 15; 10(7): e0132920 DOI: 10.1371/journal.pone.0132920 26177291 PMC4503398

[47] Ciglenecki I, Bichet M, Tena J, et al. Cholera in pregnancy: Outcomes from a specialized cholera treatment unit for pregnant women in Leogane, Haiti. PLoS Neglected Tropical Diseases. 2013 8 15; 7(8): e2368 DOI: 10.1371/journal.pntd.0002368 23967361 PMC3744413

[48] Liu L, Johnson HL, Cousens S, et al. Global, regional, and national causes of child mortality: An updated systematic analysis for 2010 with time trends since 2000. The Lancet. 2012 6 9; 379(9832): 2151–61. DOI: 10.1016/S0140-6736(12)60560-1 22579125

[49] Zupan J. Perinatal mortality in developing countries. New England Journal of Medicine. 2005 5 19; 352(20): 2047–8. DOI: 10.1056/NEJMp058032 15901857

[50] Paul VK, Sachdev HS, Mavalankar D, et al. Reproductive health, and child health and nutrition in India: Meeting the challenge. The Lancet. 2011 1 22; 377(9762): 332–49. DOI: 10.1016/S0140-6736(10)61492-4 PMC3341742 21227494

[51] Sangamam R. Perinatal mortality and morbidity among low birth weight babies. International Journal of Community Medicine and Public Health. 2017 2 4; 2(1): 51–8. DOI: 10.5455/2394-6040.ijcmph20150211

[52] Kramer MS and Victora CG. Low birth weight and perinatal mortality In: Semba RD and Bloem MW (eds.), Nutrition and Health in Developing Countries. 2nd ed. Totowa, NJ: Humana Press; 2001: 57–69. DOI: 10.1385/1-59259-225-2:57

